# High‐Strength and Tough Acid‐Base Complex Hydrogels with Memory‐Forgetting and Shape‐Memory Features

**DOI:** 10.1002/smsc.202300083

**Published:** 2023-07-26

**Authors:** Xiang Guo, Hong Wang, Xinghong Xiong, Shifang Zhao, Mei Li, Qi Chang, Jiaxi Cui

**Affiliations:** ^1^ School of Medicine Shaoxing University Shaoxing 312000 China; ^2^ Institute of Fundamental and Frontier Sciences University of Electronic Science and Technology of China Chengdu Sichuan 611731 China; ^3^ Yangtze Delta Region Institute (Huzhou) University of Electronic Science and Technology of China Huzhou 313001 P. R. China; ^4^ School of Materials Science and Engineering Qilu University of Technology (Shandong Academy of Sciences) Jinan 250353 China; ^5^ Institute of Military Training Medicine No. 2 Huaxia West Road Luoyang Henan 471031 China

**Keywords:** acid-base complex, dynamic supramolecular hydrogels, mechanical properties, memory-forgetting features, shape-memory

## Abstract

Complexes associated with strong electrostatic interactions constitute a valuable method to construct tough hydrogels, but current systems are either unstable in saline environments or lead to relatively soft matrices. Herein, a class of acid‐base complex (ABC) hydrogels made from poly(2‐(dimethylamino)ethyl methacrylate) and glassy poly(methacrylic acid) (PMA) is reported. The tough hydrogels are prepared via a dialysis‐free process and maintain their structure in saline solutions. Results indicate that the glassy feature of PMA endows the ABC hydrogels with high moduli (227 MPa), typical yielding points (3 MPa), and moderate stretchability (300% strain). The glassy backbones (PMA chains) are stiff at low temperatures, and would be melted at high temperatures, which bring about interesting shape‐memory effects and inverse memory‐forgetting behaviors. These results may inspire a simple but powerful strategy to design innovative hydrogel materials.

## Graphical abstract

High‐strength and tough acid‐base complex hydrogels make of acidic (A) and basic (B) monomers exhibit interesting shape‐memory effects and inverse memory‐forgetting behaviors because of the reversible melt of glassy backbones at high temperatures.

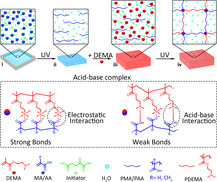

## 1 Introduction

Supramolecular hydrogels with high water content and excellent mechanical properties attract increasing attention due to their applications ranging from tissue engineering to soft robotics.^[^ ^1^ ^]^ Recently, many supermolecular hydrogels have been designed using various dynamic bonds, including hydrogen bonds, polyion complexes, hydrophobic interactions, metal coordination, host–guest, etc.^[^ ^2^ ^]^ The dissociation of these reversible interactions can dissipate great energy to ensure excellent mechanical properties^[^ ^3^ ^]^ and bring about many attractive properties, such as stimuli‐responsiveness, shape memory, processability, and environment adaptability.[^1^, ^4^] Current studies mainly focus on the contribution of dynamic bonds and relative network structures on the performance of the hydrogels, for example, introducing highly dense dynamic bonds to increase the stiffness and toughness without ultimately compromising the material's dynamic.^[^ ^5^ ^]^ Among different supramolecular interactions, the polyion complexes are strong and then widely used to design tough supramolecular hydrogels with high tensile strength and self‐healing behaviors.[^3^, ^6^] The polyion complexes used for hydrogels are often made from strong polycations and polyanions. Their preparation involves typically time‐consuming dialysis processes to remove small salt by‐products generated in complex reactions to get strong hydrogels. The obtained hydrogels are generally unstable in saline environments. We have recently found that weak acids and bases can directly form dynamic acid‐base complex (ABC) structures without forming any small salt compounds.^[^ ^7^ ^]^ Such ABC‐based hydrogels are stable in saline solutions. However, the obtained materials are relatively soft because ABC structures intrinsically equilibrate between strong electrostatic (ionic) and weak acid‐base (nonionic) states. Therefore, it is still desirable to develop ABC hydrogels with high moduli.

Here, we described a class of ABC hydrogels made from poly(2‐(dimethylamino)ethyl methacrylate) (PDEMA) and poly(methacrylic acid) (PMA). PMA has glassy backbones, which endow the ABC hydrogels with plastic‐like features and high moduli (227 MPa), typical yielding points (3 MPa), and moderate stretchability (300% strain). The glassy backbones would melt at high temperatures, which brings about interesting shape‐memory effects and inverse memory‐forgetting behaviors.

## 2 Results and Discussion

### 2.1 Preparation of Tough Hydrogels

**Figure 1:**
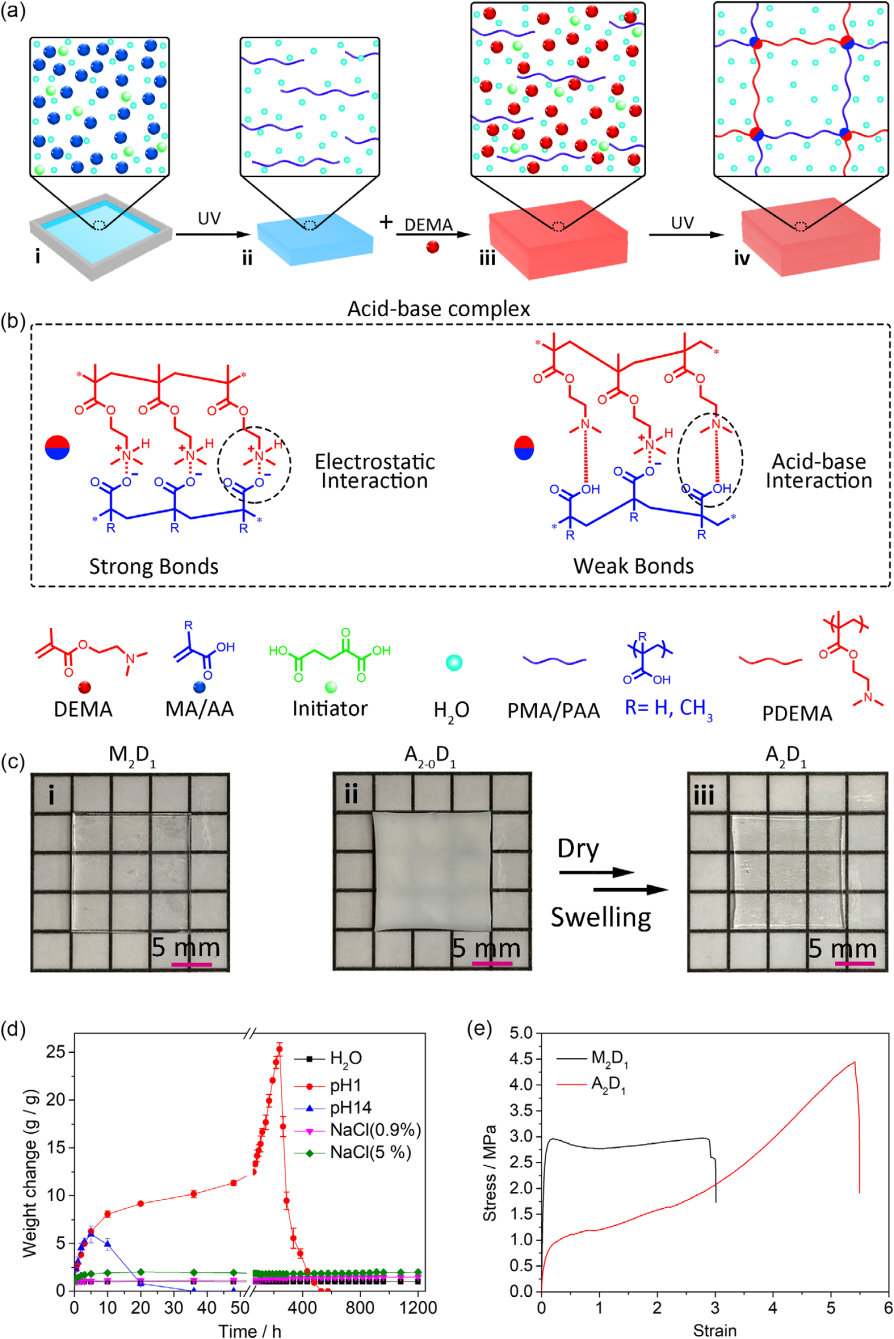
Concept and preparation of ABC hydrogels. a) Schematic illustration of the preparation process of ABC hydrogels (M_2_D_1_ or A_2_D_1_). (i) AA/MA aqueous solutions (50 wt%) containing 2‐oxoglutaric acid (photoinitiator, 0.2 mol%); (ii) PMA/PAA hydrogels; (iii) Swollen PMA/PAA hydrogels using DEMA aqueous solutions (50 wt%) containing 2‐oxoglutaric acid (photoinitiator, 0.2 mol%); (iv) DN hydrogels. b) Schematic illustration of acid‐base complex, monomer, and polymers. c) Preparation of hydrogels and corresponding photos; (i) M_2_D_1_; (ii) A_2‐0_D_1_; A_2_D_1_. d) Weight change of the ABC hydrogel (M_2_D_1_) immersed in different aqueous solutions: H_2_O, HCl solution (pH 1), NaOH solution (pH 14), saline solutions (0.9 and 5 wt%). e) Stress–strain curves of M_1_D_1_ and A_2_D_1_.

We prepared ABC hydrogels from PMA and poly(acrylic acid) (PAA). The PAA‐based system was designed as a reference without glassy backbone. **Figure** **1**a shows the preparation process of the ABC hydrogels. It is a two‐step photopolymerization process. Briefly, polyacids (PMA and PAA) were prepared at first. Afterward, quantitative DEMA solutions were added to the samples, followed by UV irradiation. The as‐prepared samples (M_2_D_0_, M_2_D_1_, A_2_D_0_, A_2_D_1_) were obtained with high polymerization yields (>91%, Figure S1, Supporting Information). Additional dialysis treatment was conducted to remove these residual monomers for the test. Different from reported polyion complexes (PIC) hydrogel, this system does not require dialysis treatment to remove counterions (NaCl) to form PIC structure and ensure excellent mechanical properity.^[6b]^ Based on simple consideration, the basic tertiary amino group of DEMA units would accept the proton of the carboxyl group to form ionic pair, which provided strong electrostatic interactions to crosslink the polymer chains. Since acrylic acids and DEMA are weak acids and bases, respectively, a dynamic equilibrium between ionic and nonionic states was expected.^[^ ^7^ ^]^ In the nonionic state, weak H‐bonds formed, which were denoted as acid‐base interaction (Figure 1b). We employed Fourier transform infrared spectroscopy (FTIR) to confirm the coexistence of both ionic and nonionic states. As depicted in Figure S2, Supporting Information, compared to the spectra of PMA and PDEMA where only strong peaks belonging to C=O stretch vibration of COOH at 1,724 cm^−1^, the ABC hydrogel made from MA and DEMA shows a new peak at 1,564 cm^−1^ that was belonged to COO^−^.^[^ ^7^ ^]^ Moreover, two peaks belonging to N‐methyl group (N(CH_3_)_2_) at 2,822 and 2,771 cm^−1^ disappeared, indicating the formation of typical electrostatic and acid‐base interactions between MA and DEMA.^[^ ^8^ ^]^ In principle, the interactions between ionic units were stronger than those formed by nonionic units (Figure 1b). Under these two kinds of supramolecular interactions, robust materials were obtained after photopolymerization. Interestingly, the as‐prepared PMA‐based hydrogels (e.g., M_2_D_1_) are transparent, while the PAA‐based hydrogels (e.g., A_2_D_1_) are opaque (Figure 1c). The opaqueness was attributed to the formation of droplet‐embedded structures during polymerization.^[^ ^9^ ^]^ SEM images of M_2_D_1_, A_2−0_D_1_, and A_2_D_1_ support this hypothesis (Figure S3, Supporting Information). The opaque sample (A_2−0_D_1_) shows obvious porous structures, indicating that water droplets existed. After polymerization, the swelling ability of the system to water decreased such that the excess water would be extruded out to form water droplets in the hydrogel matrices.^[6a]^ When such opaque samples were dried and reswelled, transparent samples were obtained.

With the sample in our hands, we first measured its glass transition temperature (*T*_g_) by rheological method. The M_2_D_1_ exhibited a high *T*_g_ (90 °C, Figure S4a, Supporting Information), indicating that the PMA‐based hydrogels are glassy at room temperature. A significantly lower *T*_g_ was observed in the PAA‐based hydrogel (8 °C, Figure S4b, Supporting Information), implying a nonglassy state at room temperature. Dynamic mechanical analysis (DMA) was further employed to study the differences. When the PAA‐ and PMA‐based hydrogels were compressed (10%), the stress of PAA‐based hydrogels was rapidly reduced at room temperature (Figure S5, Supporting Information). In contrast, in the same test conditions, the stress of PMA‐based hydrogels was reduced gradually by increasing the temperature from 25 to 90 °C. These results demonstrated that the glassy PMA backbones endowed the hydrogels with high strength. In addition, its salt stability was explored. As shown in Figure 1d, M_1_D_1_ dissolves in acidic or basic solution but maintains its weight in salt solution and pure water. In an acidic or basic solution, the equilibrium would be driven to nonionic states in which the interactions were weak, and the systems were easily dissolved. In contrast, the ABC would maintain their ionic states in saline environments; therefore, the hydrogels were stable. Figure 1e shows the typical stress–strain curves of both M_2_D_1_ and A_2_D_1_ obtained from tensile measurement. M_2_D_1_ displays a high modulus (*E*) of 35.0 ± 4.2 MPa and relatively low stretchability (maximum elongation ϵ: 2.9 ± 0.6). Moreover, a typical yielding point was observed, suggesting a plastic‐like feature. It indicated that the PMA chains in the hydrogels maintained glass states even though they were well‐solvated. In contrast, A_2_D_1_ exhibits a relatively low *E* (12.6 ± 2.3 MPa) and high ϵ (5.4 ± 0.3). These properties were attributed to the fact that flexible polymer chains facilitate the formation of more entanglement to ensure high elongation and low modulus.^[^ ^10^ ^]^ Besides high moduli, we found that M_2_D_1_ has higher water content (*C*_w_, 47.2 ± 2.2%) than A_2_D_1_ (36.3 ± 2.8%) in equilibrium saturated states. We attributed this to the different densities and strengths of hydrogen bonds.^[^ ^11^ ^]^ The high density and strength of hydrogen bonds made the PAA‐based hydrogels (A_2_D_1_) with a low water content.

### 2.2 Mechanical Performance

**Figure 2:**
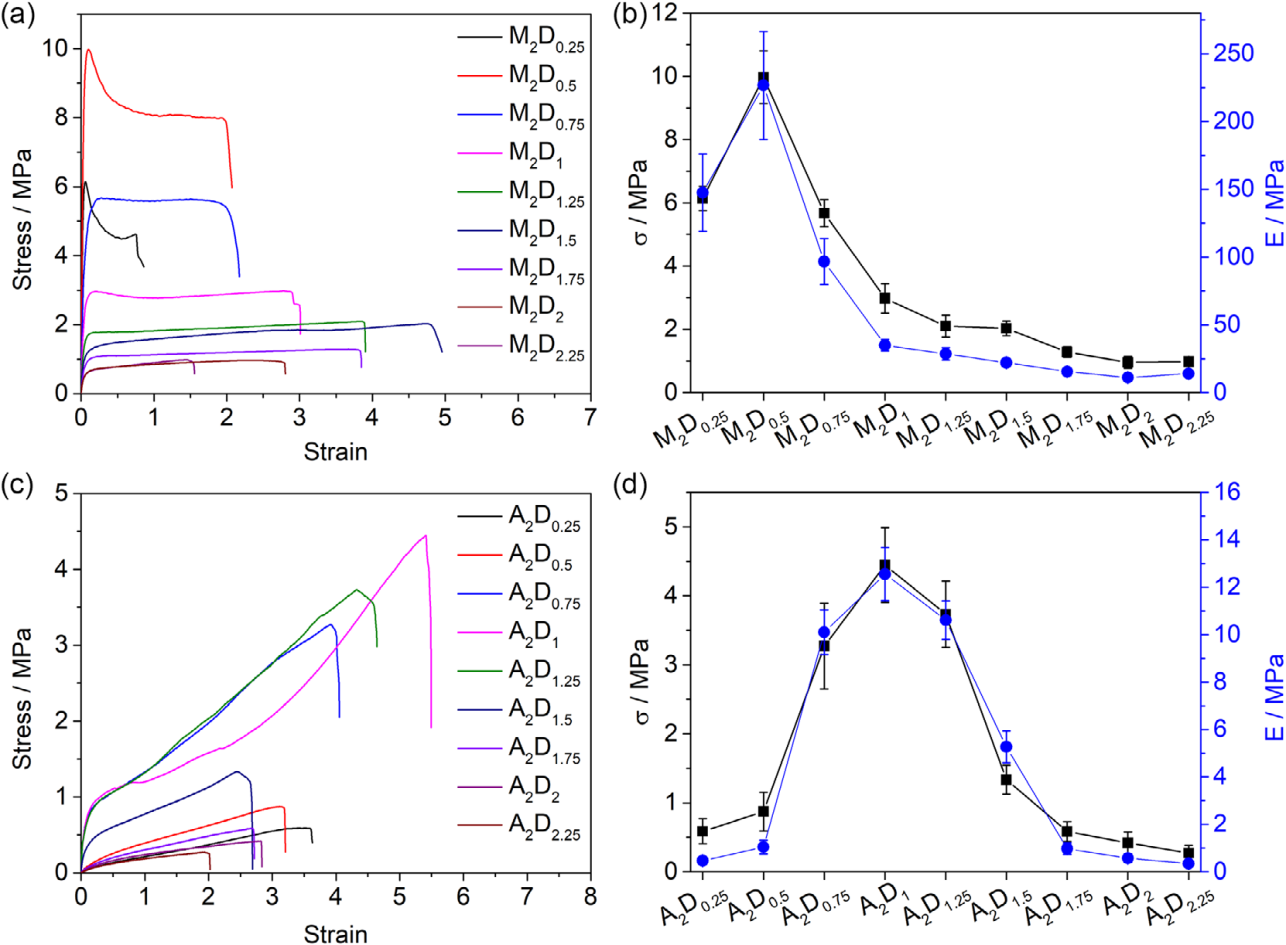
Mechanical properties of hydrogels with different acid/base molar ratios (*n*_MA_: *n*_DEMA_). a) Stress–strain curves of hydrogels with different components (*n*_MA_/*n*_DEMA_, 2/0.25–2.25). b) Fracture stress (*σ*) and Young's modulus (*E*) of the hydrogels containing different molar ratios (*n*_MA_/*n*_DEMA_, 2/0.25–2.25). The number represents the ratio of the acidic and basic monomer/composition. c) Stress–strain curves of hydrogels with containing different molar ratios (*n*_AA_/*n*_DEMA_, 2/0.25–2.25). d) Fracture stress (*σ*) and Young's modulus (*E*) of the hydrogels containing different molar ratios (*n*_AA_/*n*_DEMA_, 2/0.25–2.25). The number represents the ratio of the acidic and basic monomer/composition.

We prepared a series of supramolecular hydrogels with different components to study the structure–property relationship. With these samples in our hands, we first investigated their mechanical properties (*ε*, *E*, and strength (*σ*)) by regulating the molar ratio of monomer. As shown in **Figure** **2**, Table S1, and S6, Supporting Information, both systems of MA/DEMA and AA/DEMA show ratio‐dependent properties, but the optimized ratios were different. The highest points in both *E* and *σ* presented at *n*_MA_/*n*_DEMA_: 2/0.5. With increasing the fraction of DEMA from 2:0.5 to 2:2.25, *E* and *σ* were gradually reduced. In contrast, in the system of AA/DEMA, the highest points of *E* and *σ* presented at the ratio (*n*_AA_/*n*_DEMA_) of 2/1 (Figure 2c,d). The possible reason for this difference (the optimal ratio of the monomers) was the influence of methyl groups on the polymeric backbones. PMA had a rigid backbone, which provided good mechanical properties but also restricted the formation of ABC crosslinking structures. Therefore, the MA/DEMA systems have a smaller ratio than the AA/DEMA systems. Compared with the AA/DEMA systems (2/1), the MA/DEMA systems possess a higher ratio (2/1.5) at the best elongation.

**Figure 3:**
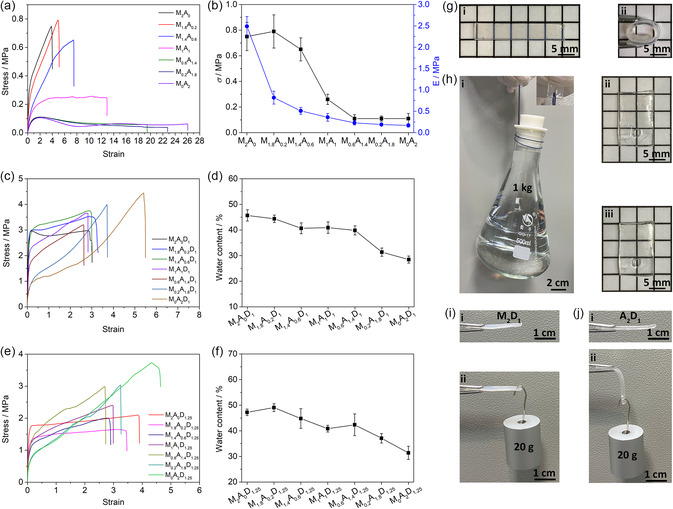
The mechanical properties of tough hydrogels. a) Stress–strain curves of hydrogels with different MA/AA molar ratios (*n*_MA_/*n*_AA_, 2/0–0/2). b) Fracture stress (*σ*) and Young's modulus (*E*) of the hydrogels containing different MA/AA molar ratios (*n*_MA_:*n*_AA_, 2/0–0/2). c) Stress–strain curves. d) Water content of different hydrogels (*n*_MA_/*n*_AA_/*n*_DEMA_, 2–0/0–2/1). e) Stress–strain curves. f) Water content of different hydrogels (*n*_MA_/*n*_AA_/*n*_DEMA_, 2–0/0–2/1.25). g) Mechanical properties of the M_2_D_1_ hydrogels: virgin (i), and curliness (ii). h) Optical images of an M_2_D_1_ specimen lifting a water bottle (1 kg, left) via a hole (i), before the lift (ii), after the lift (iii). i) Mechanical properties of the M_2_D_1_. j) A_2_D_1_ hydrogels: virgin, lift aluminum block (20 g).

The mechanical properties of the ABC supramolecular hydrogels could be finely tuned by monomer fractions. A series of hydrogels made from copolymers of AA and MA with different proportions were first prepared to demonstrate this concept. The *C*_w_ of the polymers (PAA, PMA*‐co*‐PMA, and PMA) was kept consistent (≈50%) to test mechanical properties. As shown in **Figure** **3**a,b, with increasing AA fraction, both *E* and *σ* of the hydrogels decreased, but their elongation increased gradually (Max: ≈26). On this basis, DEMA was further introduced into the AA–MA copolymers to study the performance change. As revealed in Figure 3c,d, and S7, Supporting Information, when the acid‐base molar ratio was fixed (acid/base: 2/1), a noticeable decrease in *E* and *C*_w_ was observed with AA increase. Such change should be attributed to the stiffness of methyl‐containing backbones. More MA units provided stiffer backbones to ensure higher *E* and *C*_w_. However, with increasing the AA fractions, the effect was waning because the flexible PAA polymer was favorable for forming strong electrostatic interactions to condense the networks to reduce the space for water. Therefore, when the two influences balance each other (stiffer backbones and flexible PAA polymer), the overall performance was weaker, so M_0.6_A_1.4_D_1_ has lower fracture stress than M_1_A_1_D_1_ and M_0.2_A_1.8_D_1_. In addition, similar results (Figure 3e,f, and S8, Supporting Information) were observed when additional alkaline monomers (DEMA, acid/base: 2/1.25) were added. These results indicated that the rigid polymers with methyl groups on the polymeric backbone could effectively strengthen the modulus of the supramolecular hydrogels.

The above results show a significant difference in mechanical properties between the MA/DEMA and AA/DEMA systems. The performance of M_2_D_1_ was further investigated. As depicted in Figure 3g, although M_2_D_1_ was a plastic‐like material, it could be curled without breaking. It also could carry a bottle (1 kg) without significant deformation (the holes, Figure 3h). We also explored the stiffness of these samples using force‐bearing experiments. M_2_D_1_ could load an aluminum block (20 g) without deformation (Figure 3i), while A_2_D_1_ possessed a great deformation under the same condition (Figure 3j).

### 2.3 Memory‐Forgetting Properties

**Figure 4:**
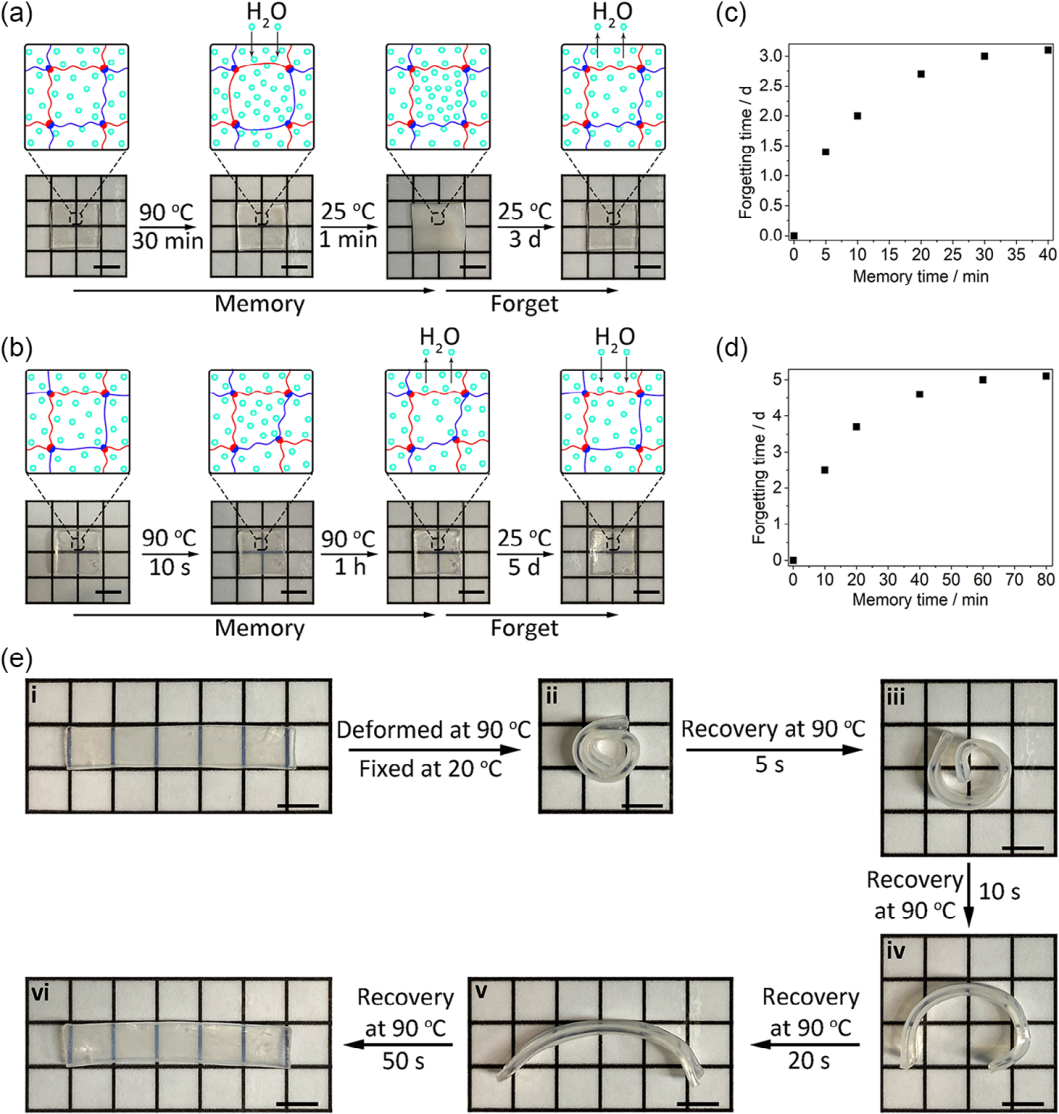
Schematic diagram of structural transformation of A_2_D_1_ a) and M_2_D_1_ b) at different temperatures and corresponding photographs. Forgetting behavior of A_2_D_1_ c) and M_2_D_1_ d) under the different memory times. e) Photos displaying the shape memory process of M_2_D_1_ at 90 °C. The sample was deformed in hot water (90 °C) and then fixed in cool water (20 °C). (i) Virgin, (ii) rolled‐up, (iii) recovery for 5 s, (iv) 10 s, (v) 20 s, (vi) 50 s. All scale bars are 5 mm.

Polymer hydrogels can take in or release water in response to temperature changes.^[^ ^12^ ^]^ The kinetics of swelling water at high temperatures could be significantly different from the water‐extrusion process at low temperatures.^[6a]^ When the temperature reduced rapidly, the polymer could quickly return to its stable state. At the same time, the absorbed water cannot be expelled from the hydrogels immediately. The excess water molecules would aggregate to form water droplets, resulting in interesting memory‐forgetting behavior. When we prepared A_2_D_1_, the as‐prepared samples were opaque. As they were allowed to dry slightly, the samples became transparent. The sample was maintained transparent even soaked in water. We thus speculated that a similar memory‐forgetting behavior existed in this system. To confirm this, we first evaluated the phenomenon of the A_2_D_1_ by observing the change of optical properties at different temperatures. As indicated in **Figure** **4**a, the transparent A_2_D_1_ (transmissivity T: 97.5 ± 0.5%) could absorb water (4.7 wt%) at high temperatures (90 °C) for 30 min, and become instantly opaque (T: 16.5 ± 1.2%) at low temperatures because of the formation of the droplets (Figure 4c and S9, Supporting Information). Such phenomenon should be attributed to the temperature‐induced asymmetric swelling and shrinking kinetics. Briefly, when the temperature decreases rapidly, the polymers could rapidly respond to heat conduction, causing the absorbed water immediately to form droplets rather than to be expelled from the hydrogels. However, this state is unstable. Over time (≈3 days), the opaque hydrogels would lose the excess water (4.6 wt%) and gradually become transparent again (Figure S9, Supporting Information, *T*: 97.1 ± 0.9%). A positive correlation between the recording and forgetting times was observed (Figure 4c), i.e., longer immersion times (memory time) at high temperature led to longer times (forgetting time) for samples to return to the original transparent state. Such a relationship indicated a memory‐forgetting feature for A_2_D_1_.

M_2_D_1_ displayed distinct behavior from A_2_D_1_. We immersed the transparent M_2_D_1_ (T : 97.8 ± 0.6%) in hot water (90 °C) for 10 s and found that the sample became subtranslucent (T : 70.8 ± 1.9%, Figure 4b). With increasing the immersion time (1 h), the sample squeezed some water (7.4 wt%) out, rather than absorbed water. At the same time, the sample became transparent (T : 97.2 ± 0.6%, Figure 4b and S10, Supporting Information). We soaked the transparent M_2_D_1_ in water at room temperature and found that it could continue to absorb the water for 5 d (3.6 wt%) without the change of transparency (Figure 4b and S10, Supporting Information). When we defined the time required for water absorption as the forgetting time, we could also get a positive correlation between memory and forgetting times (Figure 4d), suggesting a similar memory‐forgetting feature for M_2_D_1_. However, their change in transparency was inverse to A_2_D_1_. This difference could also be explained by the stiffness variation of PMA polymers at different temperatures. Briefly, PMA polymers were rigid at room temperature, against the collapse of the crosslinking network, and thus ensured more space for storing excess water. When the temperature increased (90 °C), the rigid PMA polymers were melted to form flexible chains. The flexible chains favored the formation of ABC. Polymer networks would then collapse to shrink the space. Such phase transition was fast, and the excess water would prefer to form the droplets rather than be released from the hydrogels at the first hour. Whereafter, with the temperature reduction, PMA continues to re‐form the rigid conformation, accompanied by the water's absorption.

### 2.4 Shape Memory

Recently, supramolecular hydrogels are often used to design thermoresponsive shape memory materials.^[^ ^8^, ^13^ ^]^ In principle, both permanent and temporal interactions should be constructed in these systems. The permanent interactions are used to memorize the permanent shape, whereas the weaker interactions are utilized to form temporary networks and store strain energy after shape fixing.^[13a]^ Different reversible interactions have been applied to get the shape‐memory effect, including hydrogen bonds, host–guest interactions, metal‐ligand coordination, crystallization effect, etc. However, the rigidity of the backbone has not been used for this purpose yet. Since the PMA chains could maintain their rigid feature at room temperature but melt at high temperatures, we expected a shape‐memory effort for M_2_D_1_. To confirm this idea, we rolled up a M_2_D_1_ ribbon at 90 °C and cooled the sample at 20 °C. The rolled ribbon could hold the temporal shape at room temperature. As shown in Figure 4e, when the rolled sample was immersed in hot water (90 °C), it turned back to its original shape in 50 s. In contrast, A_2_D_1_ showed viscoelastic behavior rather than a shape‐memory effect (Figure S11, Supporting Information).

## 3 Conclusion

We have prepared a class of ABC hydrogels that are mainly constructed by electrostatic interactions but stable in saline solutions. When PMA is used as the polyacid for ABC, its rigid backbone creates a plastic‐like feature. The typical sample M_2_D_1_ shows a high modulus (35.0 MPa) and a yielding point at 3 MPa, while its comparison A_2_D_1_ is relatively soft (modulus: 12.6 MPa) and elastic. The rigid backbones can restrict the collapse of polymer networks crosslinked by the interactions formed between polyacid and polybase and thus provide more space for loading water. Therefore, M_2_D_1_ even shows higher water‐swelling ability than A_2_D_1_. Moreover, the stiffness of PMA can be designed as the temporal interactions to induce interesting shape‐memory behaviors. Our results indicate that attaching small methyl groups into polymer backbones could be a powerful method to alternate hydrogels’ properties.

## 4 Experimental Section

4.1

4.1.1

##### Materials

Acrylic acid (AA), methacrylic acid (MA), 2‐(dimethylamino)ethyl methacrylate (DEMA), 2‐oxoglutaric acid (photoinitiator), and sodium chloride were purchased from Adamas. AA, MA, and DEMA were purified by column chromatography to remove the polymerization inhibitor before use.

##### Characterization

The stress–strain curves were recorded on an Instron universal testing machine (model 5943, USA) at room temperature. Their morphologies were observed with a scanning electron microscope (SEM, FEI, Inspect F50, Japan). Transmittance was recorded by UV–vis spectroscopy (Shimadzu UV1900, Japan) at room temperature (300–700 nm). Fourier transform infrared spectroscopy (FTIR) spectra were achieved using an FTIR spectrometer (Vertex80V, Germany).

##### Sample Preparation

Taking M_1_D_1_ as an example, the aqueous solution of MA (1 g, 50 wt%) containing 0.5 mol% initiator (2‐oxoglutaric acid) was irradiated by UV light (10 mW cm^−2^) for 8 h to get PMA polymers (a soft hydrogel). To the obtained sample was added an aqueous solution of DEMA (0.91 g, 50 wt%) containing initiator (0.5 mol%). The solution was completely absorbed by the PMA hydrogel in 6 h, and the obtained sample was stored for 12 h to allow the absorbed liquid to reach homogeneous distribution in the hydrogel. The swollen sample was then irradiated by UV light for 8 h, followed by 3‐day dialysis in water to equilibrate and remove residual monomers. The obtained transparent hydrogel was denoted as M_1_D_1_. The sample protocol was used to prepare other samples (M_*x*_D_*y*_, where *x* and *y* are the molar ratio of acidic and basic monomers). For other samples containing MA, AA, and DEMA, their molar ratio is *x*:*y*:*z*. Detailed sample information can be found in Table S1, Supporting Information. Note: *M* is methacrylic acid. *A* is acrylic acid. *D* is 2‐(dimethylamino)ethyl methacrylate. In this section, 3‐day dialysis is different from a dialysis‐free process.

## Conflict of Interest

The authors declare no conflict of interest.

## Supporting information

Supplementary Material

## Data Availability

The data that support the findings of this study are available from the corresponding author upon reasonable request.
