# Supplementary material for: High‐Strength and Tough Acid‐Base Complex Hydrogels with Memory‐Forgetting and Shape‐Memory Features
Source: Small Sci. 2023 Jul 26;3(9):2300083. doi: 10.1002/smsc.202300083 (PMC11936056; doi:10.1002/smsc.202300083)
Supplement: Supplementary file 1 — Supplementary Material [file SMSC-3-2300083-s001.pdf]

Supporting Information

High-strength and tough acid-base complex hydrogels with memory-forgetting and shape-memory features

*Guo Xiang, Hong Wang, Xinghong Xiong, Shifang Zhao, Mei Li, Qi Chang, and Jiaxi Cui**

Corresponding author: *E-mail: Jiaxi.Cui@uestc.edu.cn (J. Cui)

Tensile **tests**

Tensile mechanical properties were researched by the Instron universal testing machine at room temperature. The size and thickness of the specimens are determined according to the actual thickness. The strain rate is 100 mm/min. The stress-extension ratio curves were automatically recorded using the universal tester.

Rheology tests

Oscillatory experiments were implemented using a rheometer (DHR, TA Instruments). Evaporation blocker (silicone oil, 100 cst) was used to prevent solvent evaporation during the measurements. A strain sweep with oscillation frequency of 1 rad/s was conducted at the temperature range of 0-100 ℃ (heating rate: 10 ℃ / min) to determine the linear region. The strain was always controlled at 0.5%.

Dynamic mechanical analysis (DMA) tests

DMA experiments were carried out using a dynamic mechanical analysis (DMA 850). Evaporation blocker (silicone oil, 100cst) was used to prevent solvent evaporation during the

measurements. The strain was always controlled at 10 %. The stress of the samples was obtained at the temperature range of 0-100 ℃ (heating rate: 10 ℃ / min) to determine the linear region. The strain was always controlled at 10 %.

SEM tests

The samples were immersed in water for 24 h at room temperature until the sample weight did not increase anymore. They were then frozen in liquid nitrogen and lyophilized using a freeze dryer (FreeZone 2S). Finally, the frozen sample was broken and then sprayed with gold twice to ensure better imaging quality using an ion sputtering instrument (IXRF, MSP-2S). The morphology of the obtained samples was recorded using a scanning electron microscope (SEM, PHENOM PRO X).

Memory-forgetting

For A_2D_1, the as-prepared transparent A_2D_1 (A_2D_1-T) was immersed in hot water (90 °C) for 30 min. Subsequently, the hot and transparent A_2D_1 were immediately transferred to cold water (25 °C), resulting in the opaque A_2D_1 (A_2D_1-O) within 1 min. Finally, the opaque A2D1 gradually becomes transparent over time within 3 days (A_2D_1-T1).

For M_2D_1, the as-prepared transparent M_2D_1 (M_2D_1-T) was immersed in hot water (90 °C), resulting in the change from transparent to subtranslucent within 10 s. Subsequently, over time, the subtranslucent M_2D_1 gradually becomes transparent within 1 h (M_2D_1-O). Finally, upon the enhancement of time, the transparency and weight of M_2D_1 remain the same (M_2D_1-T1).

We employed the transmissivity (T), weight and photographs of A_2D_1 and M_2D_1 in the corresponding times to reveal their change process.

Shape memory

For A_2D_1, the A_2D_1 was immersed in hot water (50 °C) to deform and then fixed in cool water (5 °C) for 1 min. When the external force was removed, the A_2D_1 was retained in cool water (5 °C) for 5 min to obtain a fixed shape. Subsequently, the rolled sample was immersed in hot water (35 °C) to observe their recovery process.

For M_2D_1, the as-prepared transparent A_2D_1 was immersed in hot water (90 °C) to deform and then fixed in cool water (20 °C) for 1 min. When the external force was removed, the A_2D_1 was retained in cool water (20 °C) for 5 min to obtain a fixed shape. Subsequently, the rolled sample was immersed in hot water (90 °C) to observe their recovery process.

Table S1. Composition and physical properties of different hydrogels.

Sample	n_{MA}	n_{AA}	n_{DEMA}	C_w (%)	ε	E (MPa)
M_1A_0	1	0	0	49.3±0.3	3.9±0.5	2.5±0.2
$M_{1.8}A_{0.2}$	1.8	0.2	0	49.5±0.3	5.0±0.4	0.8±0.2
$M_{1.4}A_{0.6}$	1.4	0.6	0	49.3±0.5	7.5±0.7	0.5±0.1
M_1A_1	1	1	0	49.6±0.3	12.9±1.1	0.4±0.1
$M_{0.6}A_{1.4}$	0.6	1.4	0	49.4±0.5	19.5±1.9	0.2±0.05
$M_{0.2}A_{1.8}$	0.2	1.8	0	49.5±0.4	22.8±2.4	0.19±0.03
M_0A_2	0	2	0	49.6±0.4	26.1±3.6	0.17±0.04
$M_2D_{0.25}$	2	0	0.25	63.7±3.6	0.8±0.1	147.5±28.6
$M_2D_{0.5}$	2	0	0.5	42.3±3.7	2.0±0.4	226.6±39.9
$M_2D_{0.75}$	2	0	0.75	42.8±3.2	2.0±0.3	96.7±16.8
M_2D_1	2	0	1	47.2±2.2	2.9±0.6	35.0±4.2
$M_2D_{1.25}$	2	0	1.25	49.5±1.3	3.9±0.5	28.6±4.5
$M_2D_{1.5}$	2	0	1.5	53.1±1.6	4.8±0.6	22.1±2.1
$M_2D_{1.75}$	2	0	1.75	54.6±1.6	3.8±0.4	15.6±2.9
M_2D_2	2	0	2	58.1±1.9	2.8±0.3	14.0±2.5
$M_2D_{2.25}$	2	0	2.25	58.2±1.5	1.5±0.2	11.2±1.7
$M_{1.8}\ A_{0.2}D_1$	1.8	0.2	1	44.4±1.4	3.1±0.2	31.2±5.4
$M_{1.4}\ A_{0.6}D_1$	1.4	0.6	1	40.7±2.1	2.9±0.4	28.1±4.4
$M_1\ A_1D_1$	1	1	1	40.9±2.2	2.8±0.2	23.1±6.7
$M_{0.6}\ A_{1.4}D_1$	0.6	1.4	1	39.9±1.8	2.6±0.4	18.4±3.8
$M_{0.2}\ A_{1.8}D_1$	0.2	1.8	1	37.4±1.6	3.7±0.3	12.2±2.5
$M_0\ A_2D_1(A_2D_1)$	0	2	1	36.3±2.8	5.4±0.3	12.6±2.3
$M_{1.8}\ A_{0.2}D_{1.25}$	1.8	0.2	1.25	49.1±1.5	3.4±0.3	23.5±3.3
$M_{1.4}\ A_{0.6}D_{1.25}$	1.4	0.6	1.25	44.8±3.8	2.8±0.4	19.9±3.0
$M_1\ A_1D_{1.25}$	1	1	1.25	40.8±1.3	3.0±0.2	19.1±2.5
$M_{0.6}\ A_{1.4}D_{1.25}$	0.6	1.4	1.25	42.4±4.3	2.7±0.3	18.8±3.5
$M_{0.2}\ A_{1.8}D_{1.25}$	0.2	1.8	1.25	41.1±1.9	3.2±0.4	11.5±2.1
$M_0\ A_2D_{1.25}(A_2D_{1.25})$	0	2	1.25	42.6±3.3	4.3±0.5	10.6±3.2
$A_2D_{0.25}$	0	2	0.25	63.6±3.6	3.6±0.3	0.5±0.1
$A_2D_{0.5}$	0	2	0.5	40.7±3.4	3.1±0.4	1.1±0.3
$A_2D_{0.75}$	0	2	0.75	36.4±5.2	3.9±0.5	10.1±1.0
$A_2D_{1.5}$	0	2	1.5	45.8±4.0	2.4±0.4	5.3±0.7
$A_2D_{1.75}$	0	2	1.75	51.8±1.7	2.7±0.4	1.0±0.3
A_2D_2	0	2	2	56.8±3.5	2.8±0.3	0.6±0.2
$A_2D_{2.25}$	0	2	2.25	59.3±2.1	1.9±0.2	0.4±0.1

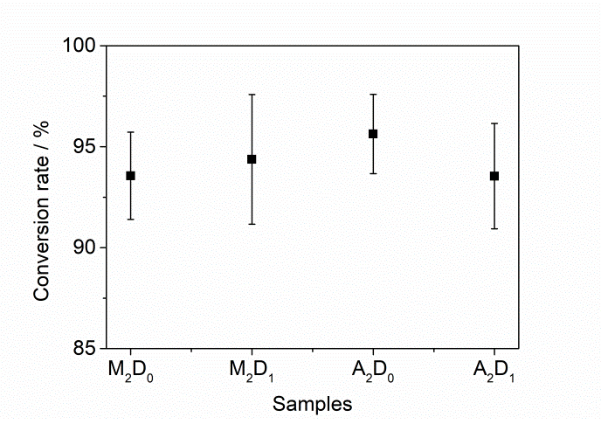

Figure S1. Monomer conversion rates of M_2D_0, M_2D_1, A_2D_0, A_2D_1.

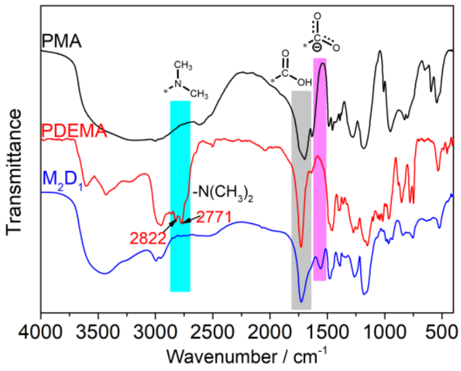

Figure S2. FTIR spectra of PMA, PDEMA and M_2D_1.

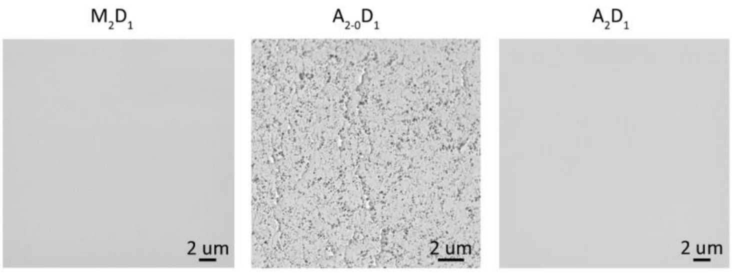

Figure S3. SEM images of M_2D_1, $A_{2\text{-}0}D_1$, and A_2D_1.

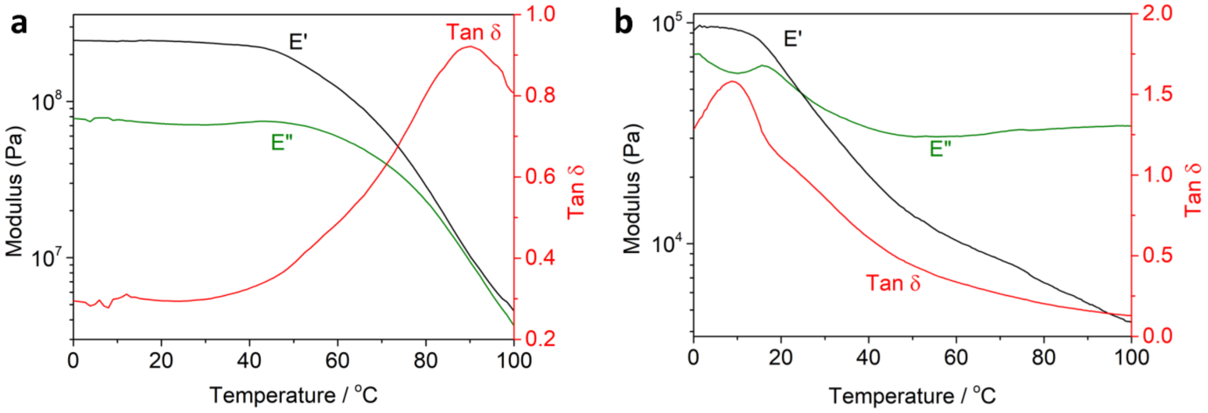

Figure S4. The storage modulus (G'), loss modulus (G''), and tan δ of M_2D_1 and A_2D_1.

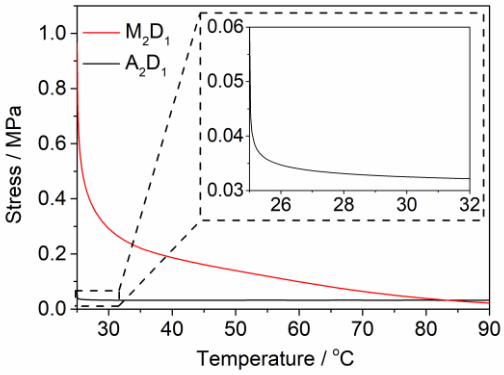

Figure S5. Dynamic mechanical analysis of M_2D_1 and A_2D_1.

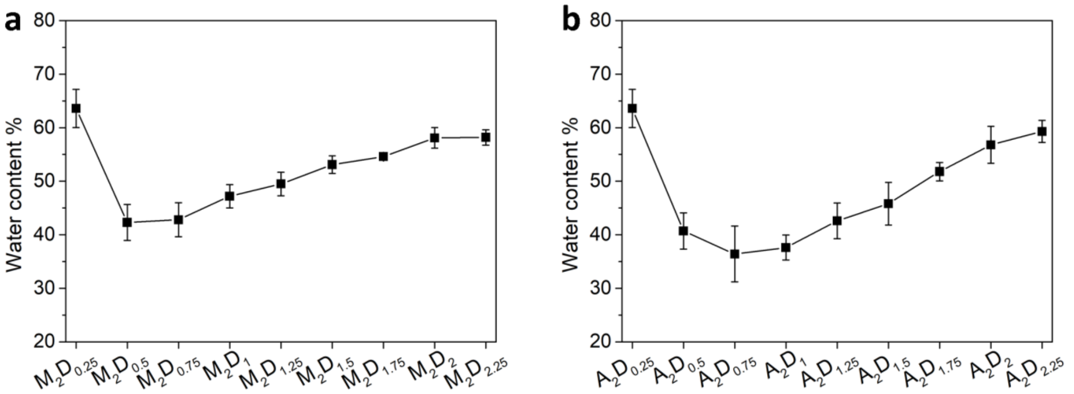

Figure S6. Water content of different hydrogels. (a): MA-DEMA ($M_2D_{0.25}$, $M_2D_{0.5}$, $M_2D_{0.75}$, M_2D_1, $M_2D_{1.25}$, $M_2D_{1.5}$, $M_2D_{1.75}$, M_2D_2, $M_2D_{2.25}$); (b): AA-DEMA ($A_2D_{0.25}$, $A_2D_{0.5}$, $A_2D_{0.75}$, A_2D_1, $A_2D_{1.25}$, $A_2D_{1.5}$, $A_2D_{1.75}$, A_2D_2, $A_2D_{2.25}$).

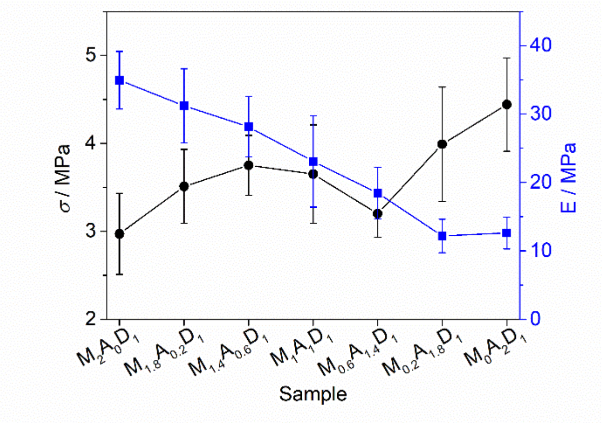

Figure S7. (b) Fracture stress (σ) and Young's modulus (E) of the hydrogels containing different mass ratios (n_{MA} : n_{AA} : n_{DEMA}).

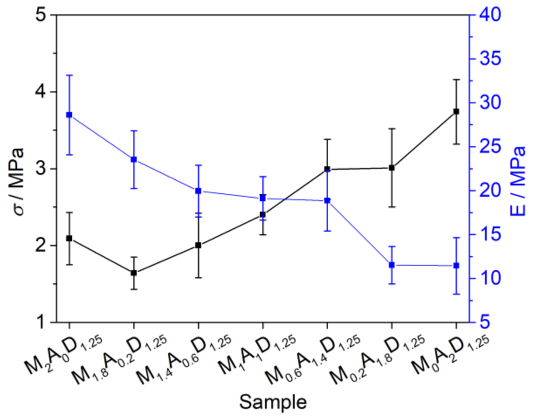

Figure S8. (b) Fracture stress (σ) and Young's modulus (E) of the hydrogels containing different mass ratios (n_{MA} : n_{AA} : n_{DEMA}).

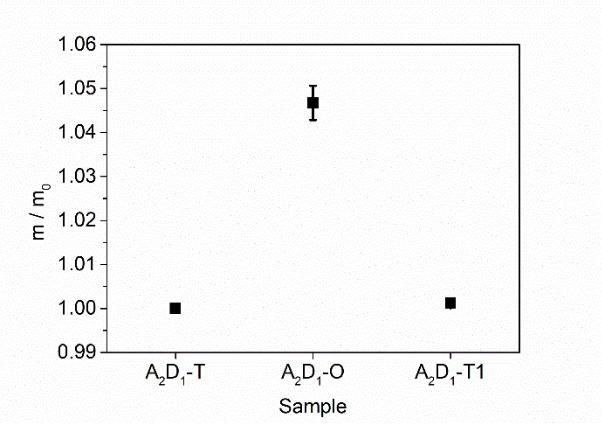

Figure S9. The weight change (m/m_0) of A_2D_1 in different states. T represents the initial transparent state. O represents the opaque state. T1 represents the transparent state through re-treating.

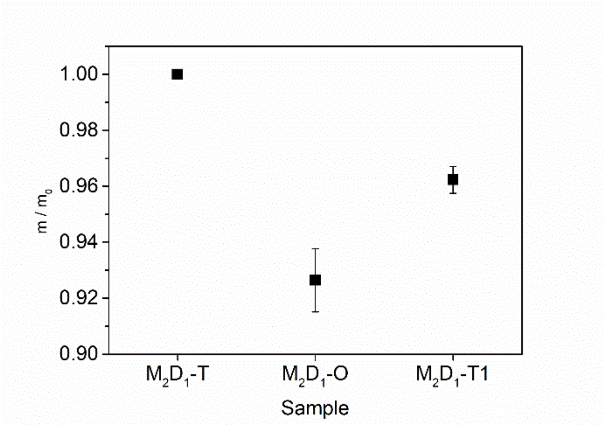

Figure S10. The weight change (m/m_0) of M_2D_1 in different states. T represents the initial transparent state. O represents the opaque state. T1 represents the transparent state through re-treating.

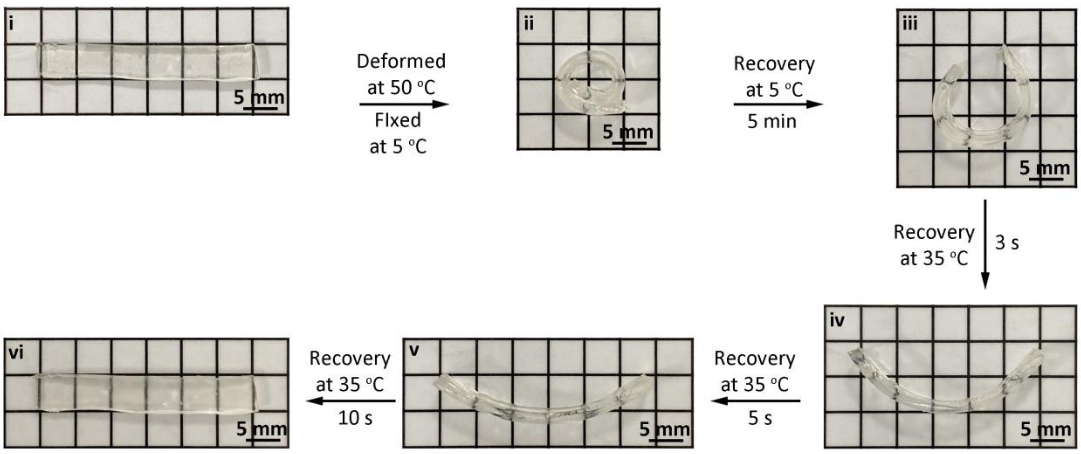

Figure S11. Photos displaying the viscoelastic behavior of A_2D_1 at 50 °C. The sample is deformed in hot water (50 °C) and then fixed in cool water (5 °C). Virgin (i), rolled-up (ii), recovery for 5 s at 5 °C (iii), recovery for 3 s at 35 °C (iv), 5 s (v), 10 s (vi).
